# Effectiveness of Psychological Interventions in Improving Relationship Functioning Among Couples Coping With Prostate Cancer: A Systematic Review and Meta‐Analysis

**DOI:** 10.1002/pon.70080

**Published:** 2025-01-13

**Authors:** Hongen Ma, Yi Yang, Yingna Li, Laura Cariola, David Gillanders

**Affiliations:** ^1^ Health in Social Science University of Edinburgh Edinburgh UK; ^2^ Department of Psychology University of Edinburgh Edinburgh UK

**Keywords:** couples, meta‐analysis, partners, prostate cancer, psycho‐oncology, psychological interventions, relationship functioning

## Abstract

**Objective:**

There is an increasing amount of literature acknowledging the significance of addressing the psychosocial impact of prostate cancer (PCa) on couples' relationship functioning and well‐being. However, research on developing and evaluating psychological interventions for individuals and couples coping with PCa remains limited. This systematic review aimed to critically evaluate and synthesise the effectiveness of psychological interventions in improving the relationship functioning of couples affected by PCa and to identify the moderating role of several methodological characteristics of intervention studies.

**Methods:**

Five databases MEDLINE, PsycINFO, Embase, Global Health, and Cochrane Library were searched up to September 2024. Twenty‐three studies with randomised trials and a total sample size of 3333 participants were included. Random effects meta‐analyses for relationship functioning, sensitivity analysis for outliers, and publication bias analysis were conducted.

**Results:**

The results showed that psychological interventions had a non‐significant trivial effect (*g* = 0.06, *p* = 0.328) on improving relationship functioning among couples coping with PCa. Subgroup analyses identified two potential moderators: firstly, the intervention format (conjoint vs. individual; *p* = 0.005), and secondly, the intervention frequency (session number < 6 vs. session number ≥ 6; *p* = 0.004).

**Conclusions:**

The findings suggest that more high‐quality intervention studies are needed to improve the relationship functioning of those affected by PCa, with screening processes to select more representative samples at entry. The implications for clinical practice highlight the need to tailor interventions to the specific needs of couples coping with PCa.

## 1 Introduction

In the UK, approximately 52,300 men are diagnosed with prostate cancer (PCa) each year [1]. While patients' survival rates and life expectancies have increased due to earlier detection and improved treatments, concerns remain regarding the chronic psychological issues associated with PCa, such as depression and anxiety [2]. It is important to note that not only patients but also their partners can be affected by these psychological consequences [3, 4]. The term ‘couple's disease’ has been coined to emphasise the impact of PCa on relationship functioning and the mental well‐being of both partners [5, 6]. Negative impacts on relationships can be observed at the early stage of diagnosis [7]. After treatment, patients may experience a range of medical complications, including erectile, urinary, and bowel dysfunction. These complications can alter their masculine self‐image, leading to feelings of shame and embarrassment [8, 9]. Consequently, these emotions may make it difficult for patients to maintain intimate relationships with their partners [10, 11].

It is also evident that partners may experience more distress than patients. After treatment, patients are more likely to rely on their partners as primary caregivers [12, 13]. Partners may feel overwhelmed while dealing with multiple stressors, such as attending to patients, dealing with uncertainty, and maintaining a work‐life balance [14, 15]. Without having those issues addressed and resolved, the relationship functioning can be disrupted, and each partner's psychosocial adaptation and well‐being will be compromised [4, 16].

Psychological interventions, such as psychosocial and psychosexual therapies, have been developed to assist individuals and couples affected by PCa [17, 18]. Empirical research has taken two different approaches to determine the effectiveness of psychological interventions in alleviating the adverse impact of PCa on relationship functioning. The first approach involves patients and partners receiving the interventions together (e.g., Refs. [4, 19]). Interventions that focus on couples tend to address dyadic adjustment and therefore may provide a more detailed examination of the impact of PCa on the patient‐partner relationship through interpersonal interactions. This approach may enhance the effectiveness of interventions by encouraging communication and shared learning within couples, thus facilitating their dyadic adjustment [20]. Another approach is to provide individual interventions for patients and partners (e.g., Refs. [21, 22]). Research has shown that patients and their partners may experience different stressors and adopt distinct coping and communication patterns [23, 24]. Therefore, interventions tailored to the individual needs of patients and partners may be more effective and allow for more flexible consultations [25].

Relationship‐focused interventions in PCa primarily target participants' communication skills, as these skills are essential for promoting dyadic adjustment and are amenable to change through psychological interventions [14]. Psychoeducation and self‐management training are also commonly included to enhance coping skills, which are critical for confronting challenges posed by PCa, such as dealing with uncertainty and making treatment decisions [26]. Although different approaches to intervention have been employed, it remains unclear which factors determine their effectiveness in improving desired outcomes, including communication, coping, and relationship functioning. A recent meta‐analysis of couple‐based interventions for couples coping with breast cancer found no statistically significant effects on dyadic relationship outcomes [27]. Similarly, another meta‐analysis reported small and non‐significant effects of couple‐based psychosocial interventions on relationship functioning across cancer types, with 33% of interventions specifically targeting couples affected by PCa [28]. While a recent review examined the effects of couple‐based psychological interventions for PCa patients and their spouses, it did not specifically explore their impact on relationship functioning [29]. As a result, there is still a gap in understanding which types of interventions are most effective in improving dyadic relationships, particularly for couples coping with PCa.

To date, no meta‐analyses have been conducted to determine the overall effect size of psychological interventions for people affected by PCa on relationship functioning, nor to explore the methodological characteristics of these intervention studies. The omission of methodological characteristics in previous systematic reviews is surprising, as information regarding intervention frequency, delivery methods (e.g., individual, conjoint), and sample characteristics (e.g., patient, partner) can offer valuable insights into whether certain design components of various studies may have contributed to conflicting results. This may help in the future development of interventions that are most beneficial to participants. With this in mind, we conducted the first systematic review with meta‐analysis to synthesise and critically evaluate the evidence of clinical trials of psychological interventions to improve relationship functioning in couples coping with PCa. The review was guided by two research questions. First, do psychological interventions significantly improve relationship outcomes for couples coping with PCa? Second, do methodological characteristics such as delivery mode, participant roles, and intervention frequency moderate the effectiveness of these interventions? By addressing these questions, this review contributes to the current knowledge by offering a more nuanced understanding of how specific intervention components can be developed to improve relationship functioning in couples coping with PCa effectively.

## 2 Methods

The review protocol was registered in the PROSPERO database (CRD42023390813). The review results were reported following the Preferred Reporting Items for Systematic Reviews and Meta‐Analyses Field [30] checklist. Ethical approval was not required.

### 2.1 Search Strategy

The database search was initially conducted in January 2023, and an updated search was performed in September 2024. The MEDLINE (1946 to 30 August 2024), PsycINFO (1806 to 30 August 2024), EMBASE (1947 to 30 August 2024), and Global Health (1973 to 30 August 2024) databases were searched through the Ovid search interface with key terms including ‘prostate’ OR ‘prostate cancer’ OR ‘prostate carcinoma’ AND ‘psychological’ OR ‘psychosocial’ OR ‘behavioural’ OR ‘psychosexual’ AND ‘intervention*’ OR ‘therapy*’ OR ‘support*’ (see Supporting Information S1: Appendix A). Cochrane Library (1996 to 2 September 2024) was also searched with mesh terms including (‘Prostatic Neoplasms’ [Mesh]) AND (‘Psychotherapy’ [Mesh] OR ‘Counselling’ [Mesh] OR ‘Psychosocial Support Systems’ [Mesh]) AND (‘Interpersonal Relations’ [Mesh] OR ‘Family Therapy’ [Mesh] OR ‘Marriage’ [Mesh]) AND (‘Spouses’ [Mesh] OR ‘Partners’ [Mesh]). In addition, relevant articles were identified by hand‐searching Google Scholar and the reference lists of the included studies.

### 2.2 Eligibility Criteria

A list of inclusion and exclusion criteria guided the selection process. Inclusion criteria set out that (1) participants were individuals diagnosed with prostate cancer and/or their intimate partners; (2) psychological interventions including psychosocial and psychosexual were delivered; (3) studies used experimental or quasi‐experimental designs, such as randomised controlled trials (RCTs) and controlled clinical trials (CCTs); (4) control groups were usual care, waitlist control, or no‐treatment control; (5) studies used validated measures that report on various aspects of relationship functioning, including relationship satisfaction, quality, and interaction; (6) studies were published in English peer‐reviewed journals. Studies were excluded regarding the following: (1) only used medical and lifestyle interventions such as exercises and physical training that focussed on physical rather than psychological outcomes; (2) nonrandomised trials (e.g., one‐group pretest‐posttest design) and case studies were excluded to ensure methodological rigour; (3) interventions involving caregivers not identified as intimate partners, such as friends and relatives.

### 2.3 Data Extraction

The search results from the database were imported into Covidence, the systematic review management software. After removing duplicates, two reviewers (H.M. and Y.L.) screened all titles and abstracts to select relevant articles. The full texts of the selected articles were then uploaded and assessed based on the inclusion and exclusion criteria. Any conflicts and ambiguities were resolved by a third reviewer (Y.Y.). The study characteristics were extracted, including the author, year of publication, participant demographics, study design, intervention content, control group, outcome measure, and main findings. For meta‐analysis, we extracted reported data on the number of participants, mean, and standard deviation of each intervention group at each data collection point. We also extracted other reported data, such as effect sizes (e.g., Cohen's *d*) and standard errors. Extraction was performed independently by two reviewers, H.M. and Y.Y., followed by a consensus check to resolve any discrepancies. A 96% agreement rate was achieved among reviewers for article selection.

### 2.4 Quality Assessment

The quality of each included study was assessed using the Effective Public Health Practice Project (EPHPP) checklist [31]. The EPHPP is a public health research assessment tool that evaluates six methodological aspects of a quantitative study: (1) selection bias, (2) design, (3) confounders, (4) blinding, (5) data collection methods, and (6) withdrawals and dropouts. Each aspect can be rated as ‘strong’, ‘moderate’, or ‘weak’. An overall rating of ‘strong’ is achieved if there are no ‘weak’ ratings, ‘moderate’ if there is one ‘weak’ rating, and ‘weak’ if there are two or more ‘weak’ ratings. The EPHPP has been evaluated as a valid and reliable quantitative study assessment tool [32] and has been used in reviews of psychological intervention studies [13, 33, 34]. Two reviewers (H.M. and Y.Y) completed the assessment individually and resolved all conflicts through discussion.

### 2.5 Data Analyses

The first step involved performing a qualitative synthesis to describe the characteristics of the included studies. Subsequently, studies with sufficient quantitative data to obtain effect sizes were selected for meta‐analyses. Relationship outcomes were measured using patient‐ and partner‐reported data, treated as distinct effect sizes. Furthermore, each intervention group was considered separately for the meta‐analysis for studies with multiple interventions. All included studies were RCTs and reported no significant baseline differences between groups, so baseline adjustment was not required. Post‐treatment means and standard deviations reported at the final time points were collected to calculate effect sizes pooled for the final meta‐analysis, considering the variation in the reported timing of the follow‐up among studies. Effect sizes were calculated using Hedges' *g*, which adjusts for small sample sizes and provides a more accurate measure than Cohen's *d* [35]. Hedges' *g* represents the standardised mean difference between the post‐treatment scores of the treatment group and the control group, corresponding to values of 0.2, 0.5, and 0.8 indicating small, moderate, and large effect sizes [36]. A random‐effects model was employed for all analyses, on the assumption of natural heterogeneity among studies. In instances where multiple outcome measures for relationship quality or satisfaction were reported in included studies, an investigation into the potential for correlated effects would be conducted. The *I* ^2^ statistic indicated low (25%), moderate (50%), and high (70%) between‐study heterogeneity. A 95% confidence level and a *p*‐value of 0.05 were selected. The analyses were performed using R (version 4.2.2) and RStudio (version 2022.12.0 + 353 for Mac) with the R meta package [37].

### 2.6 Publication Bias

The study implemented a funnel plot and Egger's regression test [38] to examine the possible existence of publication bias in the relationship functioning effects. The funnel plot displayed each effect, and an asymmetrical distribution of all effects around the averaged effect size would indicate the presence of publication bias. To test for asymmetry, Egger's regression test was conducted, which generated quantitative evidence. A significant result from Egger's test indicates the potential presence of publication bias. The objective of combining these two methods was to provide a more robust and reliable assessment of publication bias in the analysis. Subsequently, a sensitivity analysis was conducted to identify any outlying effects that may distort the pooled effect estimate. An effect is considered an outlier if there is no overlap between its confidence interval and the pooled effect's confidence interval [35]. Differences in treatment effects before and after excluding outliers were reported if any outliers were detected.

### 2.7 Subgroup Analysis

Subgroup analyses were conducted to identify moderators that contribute to the variation in the effects of psychological interventions on relationship functioning. The first analysis distinguished between individual‐based and couple‐based interventions. The second analysis compared interventions with cognitive‐behavioural components with interventions without cognitive‐behavioural components. Additionally, the study investigated the impact of intervention frequency, distinguishing between sessions less than six and those equal to or greater than six. Furthermore, subgroup analyses were conducted to examine the effects of the delivery method (in‐person vs. remote) and participant role (patient vs. partner). The existence of moderators was indicated by statistically significant differences between subgroups. All subgroup analyses adhered to the benchmark of having a minimum of four studies (*k* ≥ 4) in each subgroup [39].

## 3 Results

### 3.1 Study Selection

**FIGURE 1:**
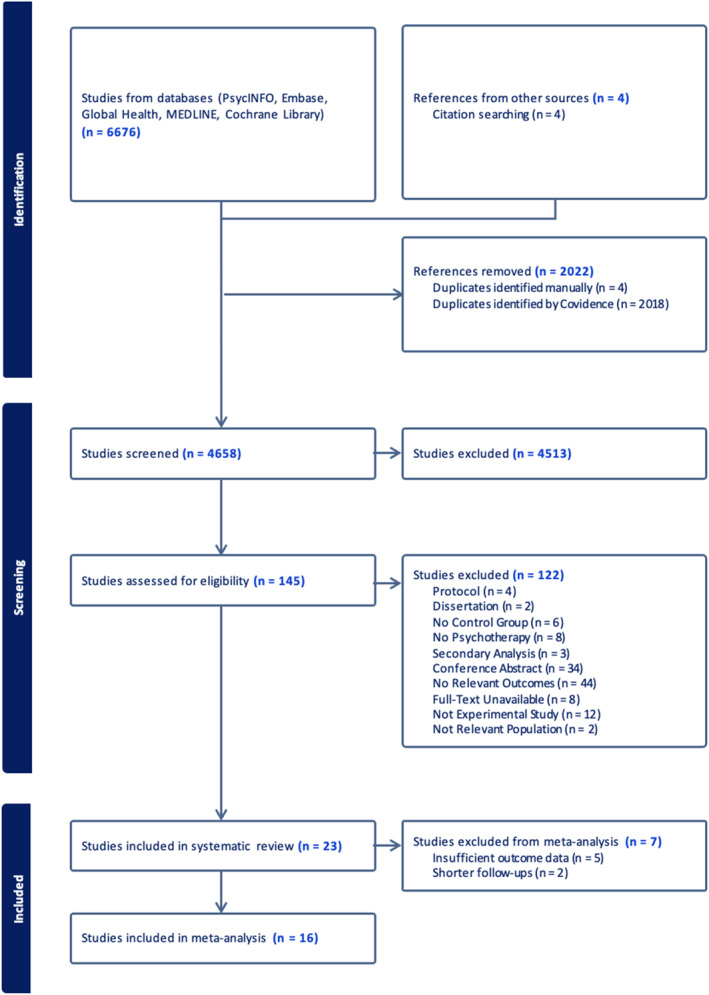
PRISMA diagram.

The PRISMA flow diagram (see Figure 1) displays information about the screening and study selection process. The results of the database search were uploaded into Covidence for screening. Overall, 6680 articles from electronic database searches and reference mining were imported. After removing duplicates, 4658 studies were screened based on their titles and abstracts. Of these, 4513 studies were excluded, leaving 145 studies with full texts assessed for eligibility. Following eligibility checks, 23 studies were included in the systematic review. The most common reasons for exclusion were conference abstracts (*n* = 34) and lack of relevant outcomes relevant to the focus of this review (*n* = 43). Of the 23 studies included, 16 had sufficient quantitative data and were therefore included in the meta‐analysis.

### 3.2 Sample Characteristics

The combined number of participants in all the studies was 3333 (see Table 1). The sample size varied from 40 to 474 individuals. Out of the 23 studies, 16 predominantly recruited couples [4, 14, 19, 40, 41, 42, 43, 44, 45, 46, 47, 48, 49, 50, 51, 52], 5 studies solely involved patients [21, 22, 53, 54, 55], and 2 studies exclusively targeted partners [25, 56]. Although one study took on caregivers as participants, it was included because 32 out of 33 caregivers identified as spouses/partners [19]. Based on the reported data, patients' mean age ranged from 60 to 73 years, while partners' mean age was between 55.7 and 63.6 years. The mean length of the relationship ranged between 27 and 39.7 years. Patients were at different stages, from pre‐diagnosis to post‐treatment. Although three studies [46, 49, 51] included same‐sex couples, the remaining studies only recruited heterosexual couples. Baseline demographic characteristics did not differ significantly among the participants across all studies. With regard to geographical location, only one study [42] was conducted in the Eastern region (the Taiwan region), while the remainder were conducted in the Western regions such as the United States, the United Kingdom, and Canada.

**TABLE 1:** Study characteristics.

Author (year)	Design	Sample size and characteristics	Theoretical frameworks	Interventions and control group	Intervention components	Number of sessions	Delivery format	Outcome measure	Included in Meta
Carlson et al. (2017)	RCT	*N* = 77 couples Pat. M age = 65.87 Par. M age = 61.70	NM	**Ind.Int**: Supportive expressive therapy **Con**: Treatment as usual	C, PS	6	In‐person	IMS	Yes
Malcarne et al. (2019)	RCT	*N* = 164 spouses Par. M age = 61.54	Bright IDEAS model	**Ind.Int**: Problem‐solving therapy **Con**: Usual psychosocial care	E	6 to 8	In‐person	DAS	Yes
Manne et al. (2004)	RCT	*N* = 60 partners Par. M age = 59.63 Rel. M length = 33.66	Stress and coping theory, cognitive and social processing theories of adaptation to difficult life events	**Ind.Int**: Psycho‐educational intervention **Con**: Usual care	E, C, PS	6	In‐person	PTGI‐relate to others	Yes
Siddons et al. (2013)	RCT	*N* = 60 patients Pat. M age = 62.34	Cognitive–behavioural framework	**Ind.Int**: Cognitive‐behavioural group intervention **Con**: Wait‐list	E, CB, C, R	8	In‐person	PCaQoL‐marital affection	Yes
Tagai et al. (2021)	RCT	*N* = 431 patients Pat. M age = 63.55	Cognitive social health information processing model	**Ind.Int**: Prostate cancer online guide & resource for electronic survivorship **Con**: Enhanced usual care	E, CB	NM	In‐person	5‐Item MIS	Yes
Giesler et al. (2005)	RCT	*N* = 99 dyads Pat. M age = 63.8	Proximal‐distal framework	**Ind.Int**: Cancer care intervention **Con**: Standard care	E	6	In‐person	DAS‐satisfaction	Yes
Osei et al. (2013)	RCT	*N* = 40 patients Pat. M age = 67.2	Transactional model of stress and coping	**Ind.Int**: Online support group **Con**: Usual care	E, PS	6	Remote	RSQ	No
Nelson et al. (2024, 2019)	RCT	*N* = 191 patients Pat. M age = 60	Acceptance and commitment therapy framework	**Ind.Int**: Acceptance and commitment therapy **Con**: Enhanced monitoring	CB	7	Mixed	SEAR	Yes (2024) No (2019)
Chambers et al. (2019, 2015)	RCT	*N* = 189 couples Pat. M age = 62.7 Par. M age = 59.78 Rel. M length = 32.48	Peer support frameworks	**Con.Int 1**: Nurse counselling intervention **Con.Int 2**: Peer support intervention **Con**: Usual care	E, SC, DS E, PS, C	6 to 7	Remote Remote	Revised‐DAS	Yes (2015) No (2019)
Couper et al. (2015)	RCT	*N* = 62 couples Pat. M age = 64 Par. M age = 61	NM	**Con.Int**: Cognitive existential couple therapy **Con**: Usual care	CB, PS	6	In‐person	FRI‐relationship function	Yes
Karlsen et al. (2021)	RCT	*N* = 35 couples Pat. M age = 63 Par. M age = 61	Social cognitive theory	**Con.Int**: Pro‐can intervention **Con**: Usual care	E, SC	6	In‐person	DAS	Yes
Lambert et al. (2022)	RCT	*N* = 33 dyads + 16 patients	Stress and coping framework, framework of dyadic coping, self‐efficacy theory	**Con.Int**: TEMPO **Con**: Wait‐list	CB, C, DS	10	Remote	Revised‐DAS	Yes
Lambert et al. (2016)	RCT	*N* = 42 couples Pat. M age = 63.7 Par. M age = 59.9 Rel. M length = 33.7	NM	**Con.Int**: Coping‐together **Con**: Minimal ethical care	E, R	1	Remote	Revised‐DAS	Yes
Manne et al. (2019)	RCT	*N* = 237 couples Pat. M age = 61 Par. M age = 57 Rel. M length = 27	Relationship intimacy model of cancer adaptation	**Con.Int**: Intimacy‐enhancing therapy **Con**: Usual care	CB, C	5	Remote	DAS	Yes
Manne et al. (2011)	RCT	*N* = 71 couples Pat. M age = 60 Par. M age = 55.7 Rel. M length = 27	Relationship intimacy model of cancer adaptation	**Con.Int**: Intimacy‐enhancing therapy **Con**: Usual care	CB, C	5	In‐person	DAS	No
Robertson et al. (2016)	RCT	*N* = 43 couples Pat. M age = 63.7 Rel. M length = 32.1	Family systems theory	**Con.Int**: Relational‐psychosexual intervention **Con**: Usual care	E	6	In‐person	SCORE‐15	Yes
Schover et al. (2012)	RCT	*N* = 115 couples Pat. M age = 64 Rel. M length = 33	Sensate focus framework	**Con.Int 1**: CARES (face‐to‐face) **Con.Int 2**: CARES (web) **Con**: Wait‐list	CB, C, E CB, C, E	3	In‐person Remote	Abbreviated‐DAS	No
Thornton et al. (2004)	RCT	*N* = 80 patients + 65 partners Pat. M age = 61.16 Par. M age = 57.23 Rel. M length = 28	NM	**Con.Int**: Communication enhancement intervention **Con**: NCCC	C	1	In‐person	DAS	No
Walker et al. (2013)	RCT	*N* = 27 couples Pat. M age = 73	NM	**Con.Int**: Educational intervention **Con**: Usual care	E	1	In‐person	DAS	No
Kemerer et al. (2023)	RCT	*N* = 68 couples Pat. M age = 68 Par. M age = 63 Rel. M length = 29	Cognitive behavioural model; mindfulness‐based cognitive model	**Con.Int 1**: Cognitive behavioural therapy **Con.Int 2**: Mindfulness therapy **Con**: Usual care	CB CB, E, R	4	In‐person	DAS‐7	Yes
Chien et al. (2020)	Quasi‐experimental with random assignment	*N* = 103 couples Pat. M age = 67.8 Par. M age = 63.6 Rel. M length = 39.7	Transactional model of stress and coping	**Con.Int 1**: Psychosocial information package **Con.Int 2**: Multimedia psychosocial intervention **Con**: Usual care	E E	6	Remote Remote	DAS‐satisfaction	Yes

Abbreviations: C = communication, CARES = Cancer Rehabilitation Evaluation System, CB = cognitive‐behavioural, Con = control, Con.Int = conjoint intervention, DAS = Dyadic Adjustment Scale, DS = decision support, E = education, FRI = Family Relationship Index, IMS = Index of Marital Satisfaction, Ind.Int = individual intervention, MIS = Marital Interaction Scale, NCCC = Norris Comprehensive Cancer Centre Standard Care, NM = not mentioned, Par = partner, Pat = patient, PCaQoL = prostate cancer‐related quality of life, PS = peer support, PTGI = Post‐Traumatic Growth Inventory, R = relaxation, RCT = randomised control trail, Rel = relationship, RSQ = Relationship Satisfaction Questionnaire, SC = support counselling, SCORE = systemic clinical outcome and routine evaluation, SEAR: Self‐Esteem and Relationship Questionnaire, TEMPO = tailored web‐based psychosocial and physical activity self‐management programme.

### 3.3 Interventions

Following a previous review [17], the intervention components across studies in this research were classified into the following categories: education (34%), communication (20%), peer support (10%), decision support (4%), relaxation (6%), supportive counselling (4%), and cognitive‐behavioural (22%). The initial six modules concentrate on sharing information, communication training, providing support, and meditation. These are distinct from the cognitive‐behavioural module, which teaches participants to work with their positive and negative thoughts and set goals to maintain behavioural changes. Several conceptual frameworks and theoretical models were mentioned across studies, such as the cognitive‐behavioural model, the relationship intimacy model of cancer adaptation, and the mindfulness‐based cognitive model. Most interventions were focussed on couples (69.6%), while a smaller percentage were either patient‐focused (21.7%) or partner‐focused (8.7%). Interventions were regularly delivered to participants either in person (60.9%) or remotely (39.1%) via phone or online websites.

### 3.4 Design of Studies

The majority of the studies were RCTs, with only one being quasi‐experimental with an intervention randomisation [42]. Five studies had multiple interventions [40, 41, 42, 46, 50]. Apart from one study [41] that had a long‐term follow‐up report extending to 5 years, the follow‐up times ranged from 3 weeks to 12 months.

### 3.5 Outcome Measures

The commonly used outcome measures for relationship functioning were the dyadic adjustment scale (DAS) and its revised (R‐DAS) and abbreviated versions (A‐DAS), which were applied in 14 studies (see Table 1). The remaining measures were the Index of Marital Satisfaction (IMS; [14]), the Post‐Traumatic Growth Inventory (PTGI) subscale related to others [56], the Marital Affection subscale of the Prostate Cancer‐Related Quality of Life Scale (PCaQoL; [55]), the 5‐item Marital Interactions Scale (MIS; [22]), the Relationship Satisfaction Questionnaire (RSQ; [54]), the Family Relationship Index (FRI; [43]), the Self‐Esteem and Relationship Questionnaire (SEAR; [21, 53]) and the Systemic Clinical Outcome and Routine Evaluation (SCORE‐15; [49]).

### 3.6 Quality of Studies

Divisional and total ratings from the EPHPP checklist were obtained (see Table 2). The overall assessment results showed that 17 studies were rated as weak ([4, 14, 21, 22, 40, 41, 44, 45, 46, 48, 49, 50, 51, 52, 53, 55, 56]), and 5 studies were rated as moderate [25, 42, 43, 47, 54], and only one study [19] was rated as strong. Examining each component individually, selection bias and blinding received the highest number of weak ratings, at 70% and 90% respectively. In contrast, the components of study design, confounders, data collection methods, and withdrawals were rated more favourably, with strong or moderate ratings in 100%, 95%, 100%, and 76% of cases, respectively.

**TABLE 2:** Quality assessment by the EPHPP.

First author (year)	Selection bias	Design	Confounders	Blinding	Data collection	Withdrawals and dropouts	Total assessment
Carlson et al. (2017)	Weak	Strong	Strong	Weak	Strong	Weak	Weak
Chambers et al. (2019, 2015)	Weak	Strong	Strong	Weak	Strong	Strong	Weak
Chien et al. (2020)	Moderate	Moderate	Strong	Weak	Strong	Strong	Moderate
Couper et al. (2015)	Weak	Strong	Strong	Moderate	Strong	Moderate	Moderate
Giesler et al. (2005)	Weak	Strong	Strong	Weak	Strong	Moderate	Weak
Karlsen et al. (2021)	Weak	Strong	Moderate	Weak	Strong	Strong	Weak
Lambert et al. (2022)	Moderate	Strong	Strong	Strong	Strong	Strong	Strong
Lambert et al. (2016)	Moderate	Strong	Strong	Weak	Strong	Moderate	Moderate
Malcarne et al. (2019)	Moderate	Strong	Strong	Weak	Strong	Strong	Moderate
Manne et al. (2004)	Weak	Strong	Strong	Weak	Strong	Strong	Weak
Manne et al. (2019)	Weak	Strong	Strong	Weak	Strong	Moderate	Weak
Manne et al. (2011)	Weak	Strong	Strong	Weak	Strong	Moderate	Weak
Osei et al. (2013)	Moderate	Moderate	Strong	Weak	Strong	Strong	Moderate
Robertson et al. (2016)	Weak	Strong	Strong	Weak	Strong	Moderate	Weak
Schover et al. (2012)	Weak	Strong	Strong	Weak	Strong	Moderate	Weak
Siddons et al. (2013)	Weak	Strong	Strong	Weak	Strong	Weak	Weak
Tagai et al. (2021)	Moderate	Strong	Strong	Weak	Strong	Weak	Weak
Thornton et al. (2004)	Weak	Moderate	Strong	Weak	Strong	Weak	Weak
Walker et al. (2013)	Weak	Moderate	Weak	Weak	Strong	Weak	Weak
Kemerer et al. (2023)	Weak	Strong	Strong	Weak	Strong	Strong	Weak
Nelson et al. (2024, 2019)	Weak	Strong	Strong	Weak	Strong	Strong	Weak

### 3.7 Publication of Bias

**FIGURE 2:**
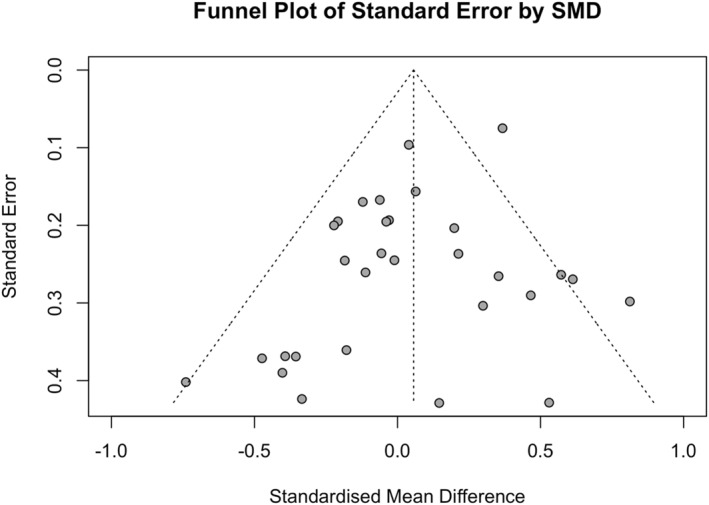
Publication bias for relationship functioning.

Based on the visual inspection of the funnel plot (*k* = 30, Figure 2), the distribution of effect sizes appears to be relatively symmetrical, indicating no potential publication bias. Furthermore, the results of Egger's test were not significant (intercept = −0.975; 95% CI [−1.98, −0.03]; *p* = 0.068). Two outliers were detected. One effect was reported by patients [44], and the other by partners [43]. The confidence intervals for both outliers were outside the overall estimated effect size's confidence interval (95% CI [−0.06; 0.17]). Although no publication bias was detected, it is important to exercise caution when interpreting the overall effect estimates due to the significant heterogeneity of the data.

### 3.8 Primary Analyses

There was no significant difference in the effects of psychological interventions on relationship functioning outcomes compared to the control conditions (*g* = 0.06, *p* = 0.328 [*k* = 30]) (see Table 3). Moderate heterogeneity was observed (*I* ^2^ = 47.1, *p* = 0.003 [*k* = 30]). After removing two identified outliers, the effect size became almost zero (*g* = 0.01, *p* = 0.846 [*k* = 28]) with low heterogeneity (*I* ^2^ = 15.1, *p* = 0.240 [*k* = 28]).

**TABLE 3:** Random effects results of meta‐analyses.

Outcome	Samples	*k*	*g*	95% CI	*p*	Heterogeneity	
						*I* ^2^	*p*
Relationship functioning	Full	30	0.06	[−0.06; 0.17]	0.328	47.1	0.003
	Exclude outliers	28	0.01	[−0.09; 0.10]	0.846	15.1	0.240

### 3.9 Subgroup Analyses

**FIGURE 3:**
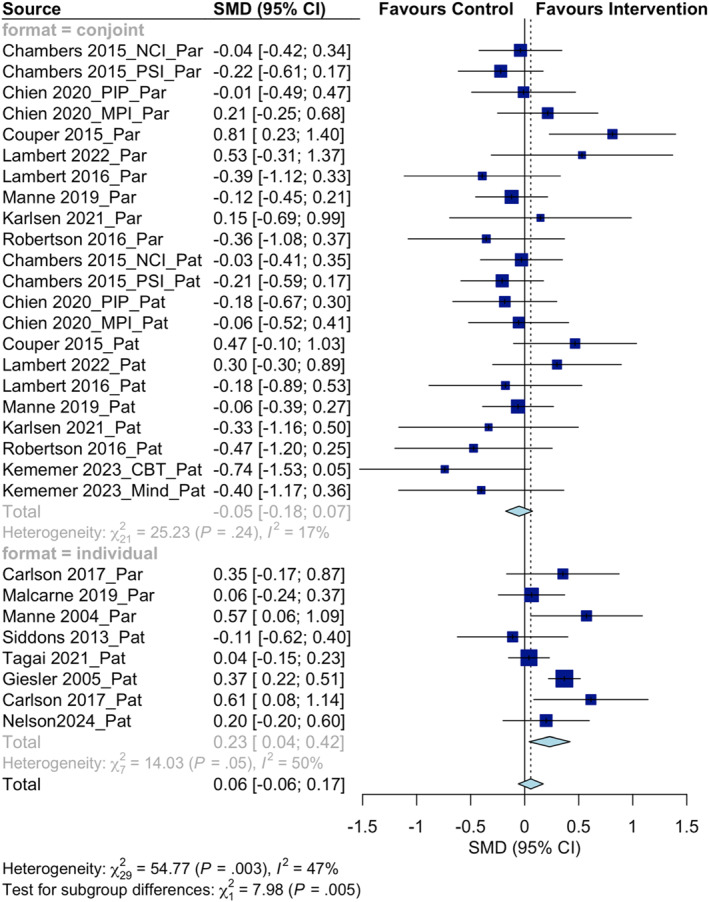
Effect sizes for relationship functioning between conjoint and individual interventions.

Potential moderators were identified through subgroup analyses (see Table 4). The first subgroup category was intervention format. The effect sizes between conjoint interventions (*g* = −0.05, *p* = 0.393 [*k* = 22]) and individual interventions (*g* = 0.23, *p* = 0.024 [*k* = 8]) showed a significant difference (*p* = 0.005), indicating that individual interventions had a larger effect and intervention format (conjoint vs. individual) moderated the effects of psychological interventions on improving relationship functioning (see Figure 3). Then, we investigated the moderating effect of the intervention delivery method by comparing in‐person and remote methods. Interventions delivered in person had an effect size of 0.14 (*p* = 0.260 [*k* = 14]), while interventions delivered remotely had an effect size of −0.04 (*p* = 0.376 [*k* = 15]). However, the difference in effect sizes was not significant (*p* = 0.161). Additionally, no significant moderation results (*p* = 0.805) were found in terms of the intervention component. Interventions with (*g* = 0.07, *p* = 0.496 [*k* = 11]) and without (*g* = 0.04, *p* = 0.548 [*k* = 19]) cognitive‐behavioural components did not significantly differ in their effect on relationship outcomes. However, the frequency of the intervention was found to be a potential moderator in the next subgroup analysis. Interventions with equal to or more than six sessions (*g* = 0.12, *p* = 0.080 [*k* = 23]) were more effective (*p* = 0.004) in improving relationship functioning compared to interventions with less than six sessions (*g* = −0.18, *p* = 0.074 [*k* = 6]). Finally, there was no significant difference (*p* = 0.583) in effect sizes between patients (*g* = 0.03, *p* = 0.718 [*k* = 17]) and partners (*g* = 0.09, *p* = 0.334 [*k* = 13]).

**TABLE 4:** Subgroup analyses.

Subgroup category	Subgroup level	*k*	*g*	95% CI	*p*	Heterogeneity		Subgroup‐*p*
						*I* ^2^	*p*	
Format	Conjoint	22	−0.05	[−0.18; 0.07]	0.393	16.8	0.237	**0.005**
	Individual	8	0.23	[0.04; 0.42]	0.024	50.1	0.051	
CBT‐inclusion	Mixed with CBT	11	0.072	[−0.16; 0.30]	0.496	44.8	0.053	0.805
	Mixed without CBT	19	0.042	[−0.10; 0.19]	0.548	49.3	0.008	
Frequency	< 6 sessions	6	−0.182	[−0.39; 0.03]	0.074	0	0.667	**0.004**
	≥ 6 sessions	23	0.119	[−0.02; 0.25]	0.080	45.7	0.010	
Delivery	In‐person	14	0.141	[−0.12; 0.40]	0.260	57.6	0.004	0.161
	Remote	15	−0.036	[−0.12; 0.05]	0.376	0	0.885	
Role	Patient	17	0.027	[−0.13; 0.19]	0.718	54.4	0.004	0.583
	Partner	13	0.092	[−0.11; 0.29]	0.334	37.6	0.083	

*Note:* Significant subgroup differences were bolded.

Abbreviation: CBT = cognitive behavioural therapy.

## 4 Discussion

The research has two primary aims: firstly, to examine the effectiveness of psychological interventions for individuals and couples coping with PCa in improving relationship functioning; and secondly, to investigate the moderating role of several methodological characteristics of included intervention studies. To estimate effect sizes and compare categorical subgroups, this research incorporated quantitative synthesis, building on previous reviews. These extensions provide insights and future considerations for designing effective psychological interventions to maximise their impact on producing desired relationship outcomes for individuals and couples dealing with PCa.

After applying inclusion and exclusion criteria, 23 intervention studies were selected, with a total sample size of 3333 individual participants. Following data extraction, 30 effect samples were used for quantitative synthesis. The meta‐analytical results showed a marginal effect of psychological interventions on relationship outcomes among individuals and couples coping with PCa. These findings are consistent with previous reviews [17, 57] which suggest that psychological interventions have not had a significant impact on improving relationship outcomes for individuals and couples affected by PCa. The reasons for this non‐significant effect are explored through several interpretations derived from the available data.

The use of small and non‐representative samples in intervention studies may limit the interpretation of results. Many studies reported that small numbers of participants reduced the statistical power to detect the effects of psychological interventions on desired outcomes (e.g., Refs. [4, 44, 45]). Furthermore, participants from different studies shared similar characteristics that could lead to ceiling effects. For example, most participants were middle‐class, white, and well‐educated [46, 48, 58]. These participants may already have access to support networks that facilitate their adjustment to PCa. Therefore, they were more likely to report high baseline scores on relationship functioning compared to PCa patients with lower socioeconomic status and education [55]. It should also be noted that a significant proportion of participants who agreed to take part in the study were in long‐term relationships. According to reported data, the average duration of relationships was over 30 years. These participants may already have good communication skills and report high relationship functioning [51]. Therefore, for participants who reported high relationship functioning at programme entry, there was little room for improvement to demonstrate the effects. Findings from a previous study showed that participants' baseline levels of distress and relationship functioning moderated the effects of treatment [48]. Although we attempted to test baseline relationship functioning as a moderator, this was not achieved as almost all included studies reported moderate to high relationship functioning at baseline, resulting in a subgroup level with insufficient numbers to meet the benchmark (*k* ≥ 4) for subgroup analysis. To achieve significant changes by reducing the risk of the ceiling effect, future studies should include a screening process to select representative samples.

Another possible reason for the trivial impact could be the use of insensitive measures. Such measures may not have the accuracy required to identify subtle changes or enhancements resulting from an intervention. If the selected measures cannot capture significant changes in the outcome of interest, the study may not be able to detect the effects of the intervention. For example, one study indicated that the SCORE‐15 may lack sensitivity as a measure of relationship functioning and should be substituted with other measures such as the DAS in the future [49]. The DAS has been reported to have high sensitivity through evaluation studies [59, 60], while the SCORE‐15 is still being evaluated to establish its clinical cut‐off points [61]. Some studies have used subscales with narrow score ranges [22, 55, 56], which may hinder the ability to detect changes in desired relationship functioning outcomes. Hence, future studies should use relationship functioning measures with strong psychometric features.

The high level of heterogeneity between studies may also have contributed to the non‐significant results. Limited research on the psychosocial impact of PCa in individuals and couples means there is no comprehensive knowledge for designing the most effective psychological interventions to improve their relationship functioning. Therefore, many of the studies included in this meta‐analysis were pilot studies with exploratory purposes. As a result, there was a high degree of heterogeneity in terms of differences in treatment components, samples, data analysis methods, and outcome measures. This heterogeneity was statistically significant, which emphasises the need for caution when interpreting the results of this meta‐analysis.

Following the identification of heterogeneity among studies, several subgroup analyses were conducted to determine which subgroups of participants benefited most from psychological interventions. Our findings indicate that participants benefited more from individual interventions than conjoint interventions. This highlights the complexity of designing interventions for couples coping with PCa. Couple‐focused interventions traditionally use the conjoint approach due to the benefits of involving both patients and partners, such as open communication and shared learning opportunities. However, this approach can present barriers to recruiting larger and more representative samples [25]. As previously mentioned, recruiting participants for clinical trials is a common challenge. Over two‐thirds of included studies implemented conjoint interventions. Many of these reported low recruitment and high attrition (e.g., Refs. [48, 52, 54]). One possible explanation is that couples coping with PCa may have found it difficult and uncomfortable to discuss their differing perspectives on the importance of sexual dysfunction. For instance, partners often preferred to accept erectile dysfunction (ED), while patients viewed it as harmful to intimacy [4]. Also, partners were found to report mild to severe anxiety due to patients' psychological distress and sexual dysfunction [62]. This may intensify relationship tensions and complicate joint interventions, particularly for couples who are already facing challenges such as poverty, mental health issues, or the demands of parenting. As a result, couples may experience relationship disruption during conjoint interventions, leading them to drop out of studies. As for couples who completed the trials, they may already have better communication skills upon entering. Therefore, these issues ultimately result in small and unrepresentative samples in conjoint interventions. In contrast, individual interventions offer a more flexible environment without imposing stressful commitments on patients and partners, as they can have their specific needs addressed. Compared to conjoint interventions, individual interventions may have a higher uptake rate with a lower risk of a ceiling effect.

We also identified the frequency of the intervention as a potential moderator in addition to the intervention format. The significant difference between subgroups suggests that the overall impact of psychological interventions on relationship functioning may vary depending on the number of sessions. Interventions with fewer than six sessions may be more time‐efficient and accessible for participants with busy schedules compared to interventions with six or more sessions. However, shorter interventions may have limited impact, particularly for complex issues such as relationship functioning. It takes time to fully comprehend the psychosocial impact of PCa. Therefore, longer interventions may provide sufficient time for participants to explore their challenges through extensive and ongoing training. However, it is important to exercise caution when making decisions based on observations of a significant difference between subgroups but non‐significant effects within each subgroup. There might be some confounders explaining the subgroup differences. Future studies with larger data sets may be able to systematically evaluate other aspects of intervention dosage, such as the proportion and total duration of contact, to make informed recommendations.

Although no moderating effects of the remaining methodological characteristics were found, some recommendations may be useful for guiding future intervention studies. First, the use of online‐based interventions has increased since the start of the Covid‐19 pandemic. Most studies conducted during the pandemic were delivered remotely (e.g., Refs. [19, 22, 42]). Although no differences in effects were observed, online interventions may be more economical and flexible compared to face‐to‐face interventions. However, more cost‐effectiveness analysis is needed when designing psychosocial interventions for individuals and couples affected by PCa in the future. Previous research suggested that interventions were more beneficial for partners [17, 57]. However, our study found no significant improvements in relationship functioning for either patients or partners after treatment. The non‐significant results may be due to ceiling effects observed in the included studies. Future studies should aim to recruit more distressed couples with low relationship functioning at baseline, while also identifying factors such as economic hardship, family strain, or long‐standing marital conflict, to better understand how these issues may influence intervention outcomes. Overall, it is important to keep in mind that subgroup analyses in meta‐analyses are exploratory and do not provide causal evidence [63].

### 4.1 Limitations

This study is the first known to use quantitative synthesis to examine the effectiveness of psychological interventions on relationship functioning for couples coping with PCa. While the study has strengths, it also has limitations that should be addressed when interpreting the results. The list of evaluated methodological features of intervention studies through subgroup analyses is not comprehensive. Subgroup comparisons were not possible due to the limited number of studies for certain characteristics. For example, baseline relationship functioning could not be included as a moderator because most studies reported moderate to high relationship functioning, making subgrouping unattainable. Also, moderator analyses for variables such as age, cancer stage, and cancer continuum were not conducted due to the limited variability and the relatively small number of included studies, which would have resulted in subgroups too small to produce reliable results. Future research with larger datasets should explore how these variables affect intervention outcomes. Finally, some subgroup analyses had unbalanced sample size distributions. The subgroup of individual interventions (*k* = 8) and the subgroup of interventions with less than 6 sessions (*k* = 6) just passed the minimum requirement for the subgroup analysis (*k* ≥ 4). This may lead to a lack of statistical power, a common issue in subgroup analyses [64].

Although we found no significant difference between studies with CB‐based components and those without, we were unable to isolate the effects of specific intervention components. Most interventions combined clinical (e.g., CB‐based) and non‐clinical (e.g., educational or communication skills) components, and key details (e.g., the proportion of CB‐based components in each study) were often missing. As a result, our findings reflect the overall effectiveness of psychological interventions but do not allow for conclusions about the relative effectiveness of individual components. Additionally, the lack of overlapping interventions and shared comparators made it impractical to apply network meta‐analysis (NMA) in this review. Future research should address these limitations by designing studies that examine well‐defined interventions, such as CB‐based therapies (e.g., trauma‐informed CBT or cognitive processing therapy). Methods like NMA could then be used to compare the relative effectiveness of distinct intervention types. This would provide greater clarity on the most effective approaches for improving relationship functioning among couples coping with cancer.

Another limitation of this study is that most of the included studies were of low quality, as indicated by our study quality assessment. Although the EPHPP checklist provides a comprehensive framework for assessing study quality in this review, it is important to interpret the assessment results with caution. First, it should be recognised that criteria such as blinding may not be fully applicable to psychosocial interventions. Blinding can be a challenge in these trials because participants and interventionists are usually aware of the intervention, unlike in pharmacological trials [65]. In addition, psychosocial interventions may face unique selection biases due to factors such as stigma and participant motivation [66]. Therefore, despite the low quality of most study components in this review, these results should be interpreted in the context of the specific characteristics of psychosocial studies. Besides, only a small number of studies with low quality were expected due to the limited research in the field. While it is acceptable to conduct a quantitative synthesis combining low‐quality studies, caution must be exercised when including and interpreting the results of these studies. Analyses were conducted to detect the level of heterogeneity. Following the detection of significant heterogeneity, sensitivity and subgroup analyses were then performed to identify outliers and assess different study characteristics that may account for differences in effect sizes. Given the imbalance in study quality ratings, a meta‐regression to examine the association between effect sizes and study quality was deemed impractical and, therefore, was not performed. Hence, future studies with a more balanced distribution of study quality ratings are encouraged to conduct meta‐regression analyses to explore the potential association between effect sizes and study quality.

Finally, only studies with randomised trials were included in this review. Although RCTs are considered the gold standard of evidence in systematic reviews and meta‐analyses [63], they often have some practical limitations in intervention studies. For example, it has been mentioned that it was not possible to blind participants due to intervention conditions and ethical considerations [19, 43, 45]. Therefore, to provide a comprehensive review of the effects of psychological interventions, future studies should include a broader representation of studies by including both RCTs and non‐RCTs. Finally, our review is specific to PCa. Some recent systematic reviews and meta‐analyses of psychosocial interventions in other cancer populations, such as breast cancer, have also found no significant overall effect of psychosocial interventions on improving relationship functioning between patients and their partners [27, 28]. Therefore, future research should explore the applicability of these findings to other cancer populations. Comparative studies across different cancer types could further demonstrate the generalisability of psychosocial interventions and identify the specific needs of different patient groups. In addition, most studies were conducted in Western regions and primarily focussed on heterosexual couples, limiting the applicability of the findings to other cultural or relationship contexts. Future research should include diverse geographical locations and relationship types, such as same‐sex couples, to enhance the generalisability of the findings.

### 4.2 Clinical Implications

For health practitioners working with individuals and couples affected by PCa, our systematic review suggests that individual interventions may offer enhanced benefits for improving relationship functioning compared to conjoint interventions. Individual interventions, tailored to participants' unique needs and offering flexible engagements, show higher uptake rates among more representative samples. Additionally, interventions featuring longer sessions (i.e., six or more sessions) may provide sufficient time to address the complex psychosocial impact of PCa on relationship functioning. To maximise the impact of psychological interventions, practitioners should consider selecting participants who exhibit distress and low relationship functioning at entry, while also being mindful of potential limitations in the available evidence.

## 5 Conclusion

The findings of this systematic review contribute to the current literature on using psychological interventions to improve relationship functioning among couples coping with PCa. Although previous interventions did not significantly impact relationship functioning, recruiting more representative samples of participants with high distress and low relationship functioning may lead to substantial effects in future studies. Identified potential moderators through subgroup analyses may assist practitioners in developing future effective interventions by using individual formats and longer sessions. Nevertheless, more clear indications are required as research into the application of psychological interventions for individuals affected by PCa is still limited. Outlined limitations in this study emphasise the necessity for additional high‐quality research to investigate further methodological characteristics and study design that could impact the effect's magnitude.

## Conflicts of Interest

The authors declare no conflicts of interest.

## Supporting information

Supporting Information S1

Supporting Information S2

Supporting Information S3

Supporting Information S4

## Data Availability

The data that supports the findings of this study are available in the supplementary material of this article.
