# Supplementary material for: Effectiveness of Psychological Interventions in Improving Relationship Functioning Among Couples Coping With Prostate Cancer: A Systematic Review and Meta‐Analysis
Source: Psychooncology. 2025 Jan 13;34(1):e70080. doi: 10.1002/pon.70080 (PMC11728261; doi:10.1002/pon.70080)
Supplement: Supplementary file 1 — Supporting Information S1 [file PON-34-e70080-s001.docx]

**Database:**

Cochrane Library

Search Name: September 2014 Search

Date Run: 02/09/2024 16:19:27

Comment:

ID Search Hits

#1 MeSH descriptor: [Prostatic Neoplasms] explode all trees 8918

#2 MeSH descriptor: [Psychotherapy] explode all trees 36007

#3 MeSH descriptor: [Psychosocial Support Systems] explode all trees 111

#4 MeSH descriptor: [Counseling] explode all trees 7629

#5 #2 OR #3 OR #4 42271

#6 MeSH descriptor: [Interpersonal Relations] explode all trees 8879

#7 MeSH descriptor: [Marital Therapy] explode all trees 133

#8 MeSH descriptor: [Family Therapy] explode all trees 1188

#9 MeSH descriptor: [Spouses] explode all trees 512

#10 MeSH descriptor: [Family Characteristics] explode all trees 1813

#11 MeSH descriptor: [Spouses] explode all trees 512

#12 #6 OR #7 OR #8 OR #9 OR # 11 OR #11 488597

#13 #1 AND #5 AND #12 37

**Database:**
APA PsycInfo <1806 to August 2024 Week 5>
Embase Classic+Embase <1947 to 2024 August 30>
Global Health <1973 to 2024 Week 35>
Ovid MEDLINE(R) and Epub Ahead of Print, In-Process, In-Data-Review & Other Non-Indexed Citations, Daily and Versions <1946 to August 29, 2024>

| **#** | **Query** | **Results from 2 Sep 2024** |
| --- | --- | --- |
| 1 | 'prostate cancer'.mp. [mp=ti, ab, hw, tc, id, ot, tm, mf, tn, dm, dv, kf, fx, dq, bt, cw, nm, ox, px, rx, ui, sy, ux, mx] | 472,908 |
| 2 | 'psychological'.mp. [mp=ti, ab, hw, tc, id, ot, tm, mf, tn, dm, dv, kf, fx, dq, bt, cw, nm, ox, px, rx, ui, sy, ux, mx] | 2,315,954 |
| 3 | 'psychosocial'.mp. [mp=ti, ab, hw, tc, id, ot, tm, mf, tn, dm, dv, kf, fx, dq, bt, cw, nm, ox, px, rx, ui, sy, ux, mx] | 499,477 |
| 4 | 'intervention*'.mp. [mp=ti, ab, hw, tc, id, ot, tm, mf, tn, dm, dv, kf, fx, dq, bt, cw, nm, ox, px, rx, ui, sy, ux, mx] | 4,509,988 |
| 5 | 'therapy*'.mp. [mp=ti, ab, hw, tc, id, ot, tm, mf, tn, dm, dv, kf, fx, dq, bt, cw, nm, ox, px, rx, ui, sy, ux, mx] | 17,746,706 |
| 6 | 'behavioral'.mp. [mp=ti, ab, hw, tc, id, ot, tm, mf, tn, dm, dv, kf, fx, dq, bt, cw, nm, ox, px, rx, ui, sy, ux, mx] | 1,393,638 |
| 7 | 'support'.mp. [mp=ti, ab, hw, tc, id, ot, tm, mf, tn, dm, dv, kf, fx, dq, bt, cw, nm, ox, px, rx, ui, sy, ux, mx] | 14,129,342 |
| 8 | 4 or 5 or 7 | 31,884,343 |
| 9 | 2 or 3 or 6 | 3,837,063 |
| 10 | 1 and 8 and 9 | 6,639 |

'prostate cancer'.mp. [mp=ti, ab, hw, tc, id, ot, tm, mf, tn, dm, dv, kf, fx, dq, bt, cw, nm, ox, px, rx, ui, sy, ux, mx]
'psychological'.mp. [mp=ti, ab, hw, tc, id, ot, tm, mf, tn, dm, dv, kf, fx, dq, bt, cw, nm, ox, px, rx, ui, sy, ux, mx]
'psychosocial'.mp. [mp=ti, ab, hw, tc, id, ot, tm, mf, tn, dm, dv, kf, fx, dq, bt, cw, nm, ox, px, rx, ui, sy, ux, mx]
'intervention*'.mp. [mp=ti, ab, hw, tc, id, ot, tm, mf, tn, dm, dv, kf, fx, dq, bt, cw, nm, ox, px, rx, ui, sy, ux, mx]
'therapy*'.mp. [mp=ti, ab, hw, tc, id, ot, tm, mf, tn, dm, dv, kf, fx, dq, bt, cw, nm, ox, px, rx, ui, sy, ux, mx]
'behavioral'.mp. [mp=ti, ab, hw, tc, id, ot, tm, mf, tn, dm, dv, kf, fx, dq, bt, cw, nm, ox, px, rx, ui, sy, ux, mx]
'support'.mp. [mp=ti, ab, hw, tc, id, ot, tm, mf, tn, dm, dv, kf, fx, dq, bt, cw, nm, ox, px, rx, ui, sy, ux, mx]
4 or 5 or 7
2 or 3 or 6
1 and 8 and 9


<https://www.ezproxy.is.ed.ac.uk/login?url=http://ovidsp.ovid.com/ovidweb.cgi?T=JS&NEWS=N&PAGE=main&SHAREDSEARCHID=5wOik9x4VBnow8ACD7924j484xsAQu68SmPDPbqqSdREEqOoWW76PdblpJQ1kv7NT>
